# Bacteria Co-colonizing with *Clostridioides Difficile* in Two Asymptomatic Patients

**DOI:** 10.1515/biol-2019-0071

**Published:** 2019-12-31

**Authors:** Wei Hong, Jing Yang, Yumei Cheng, Xiaolin Huang, Fengqin Rao, Ting Zhang, Pixiang Wang, Jian Liao, Xiaolan Qi, Zhizhong Guan, Zhenhong Chen, Guzhen Cui

**Affiliations:** 1 Key Laboratory of Endemic and Ethnic Diseases, Guizhou Medical University, Ministry of Education, Guiyang 55004, Guizhou, China; 2 Key Laboratory of Medical Molecular Biology, Guizhou Medical University, Guiyang 55004, Guizhou, China; 3 Guiyang Maternal and Child Health Hospital, Guiyang 550004, Guizhou, China; 4 Department of Critical Care Medicine, the Affiliated Hospital of Guizhou Medical University, Guiyang 550004, Guizhou, China; 5 School/Hospital of Stomatology of Guizhou Medical University, Guiyang 550004, Guizhou, China; 6 Department of Biosystems Engineering, Auburn University, Auburn, Alabama 36849, United States; 7 School of Basic Medical Science, Guizhou Medical University, Guiyang, 550025, China Guiyang 550025, Guizhou, China

**Keywords:** *Clostridium difficile* infection, asymptomatic patients, co-colonization, microbial diversity, 16S rDNA sequencing

## Abstract

**Background:**

*Clostridium difficile* infection (CDI) is the leading cause of nosocomial diarrhea. Co-colonization of key bacterial taxa may prevent the transition from asymptomatic *C. difficile* colonization to CDI. However, little is known about the composition of key bacterial taxa in asymptomatic patients.

**Methods:**

In the present study, the culture method was used to examine the composition of stool microbiota in two asymptomatic patients from Guizhou, China.

**Results:**

A total of 111 strains were isolated and phylogenetic relationships were determined by 16S ribosomal gene sequencing and Molecular Evolutionary Genetics Analysis version 7. The results demonstrated that *Escherichia* (33.3%, 37/111), *Clostridium* (24.3%, 27/111) and *Enterococcus* (11.7%, 13/111) exhibited a high ratio in asymptomatic patients. These isolates derived from two phyla: *Firmicutes* (51.3%, 57/111) and *Proteobacteria* (44.1%, 49/111). In addition, co-colonization of human pathogens *Fusobacterium nucleatum*, *Ralstonia pickettii*, *Klebsiella pneumoniae*, *Klebsiella quasipneumoniae* and *Clostridium tertium* with *C. difficile* was identified. To the best of our knowledge, these pathogens have not been co-isolated with *C. difficile* previously.

**Conclusions:**

In summary, the present study identified the composition of fecal microbiota in two asymptomatic patients in Guizhou, China. These results suggested that co-infection with human pathogens may be ubiquitous during CDI progression.

## 1 Introduction

*Clostridium difficile*, recently renamed *Clostridioides difficile* [1], is a gram-positive, rod-shaped and strictly anaerobic human pathogen. *C. difficile* infection (CDI) is the leading cause of nosocomial diarrhea, which poses a major threat to health care facilities, including long-term care facilities, nursing homes and hospitals worldwide [2, 3]. The clinical symptoms of CDI range from mild diarrhea to pseudomembranous colitis, which may result in death.

The mechanism of CDI onset is associated with antibiotic usage. Antibiotics are used to treat bacterial infections; however, they disrupt the integrity of the intestinal microbiota in the human gut. The niche created by antibiotics provides a competing advantage to *C. difficile* against probiotics, thus leading to the propagation of *C. difficile* and overproduction of toxin A and toxin B [4]. Toxin A (enterotoxin) and toxin B (cytotoxin) induce cell death, inflammation and the accumulation of neutrophils, which result in various symptoms of CDI [4].

Following a course of antibiotic therapy for CDI, the recurrence of CDI has been described in 10-30% of patients after first infection and up to 60% after multi-episode infections [5]. Furthermore, recurrent CDI (RCDI) leads to increased morbidity and mortality [6], thus, the treatment of RCDI is still challenging. In recent years, fecal microbiota transplantation (FMT), which transfers healthy fecal microbiota from a healthy donor to a patient with RCDI, has been demonstrated to be effective in treating RCDI with an effective rate of ~90% [6, 7]. These results suggested that the integrity of the gut microbiota be key for the treatment of CDI and RCDI.

By using whole metagenome shotgun sequencing, Vincent et al. demonstrated that co-colonization with key bacterial taxa may prevent the increased proliferation of *C. difficle* [8]. In clinical practice, a number of asymptomatic patients with *C. difficile* colonization do not develop CDI. The present study hypothesized that the presence of certain microbes in these patients may serve a pivotal role in preventing the transition of asymptomatic colonization of *C. difficile* to CDI. Thus, these asymptomatic patients may be used as an appealing gut microbial homeostasis model in the nosocomial environment. Study of the composition of gut microbiota in this model may help develop treatments for CDI/RCDI. In addition, the intestinal microbial community in asymptomatic patients is easier to study compared with that of the healthy human fecal microbiome, as it contains lower bacterial diveristy and retains pivotal information.

Although the gut microbiota composition of *C. difficile* asymptomatic carriers may be important for finding new CDI/RCDI treatment strategies, limited information is available regarding the bacteria that co-colonize with *C. difficile* in the asymptomatic patients. In developing countries, the awareness of CDI is insufficient, and the dietary habits are distinct from North America and Europe. The present study used the culture method to study the diversity of microbes in two *C. difficile* asymptomatic patients in Guizhou, China.

## 2 Methods

### 2.1 Selection criteria and ethics

Consecutive patients who were admitted to ICU wards of affiliated hospital of Guizhou Medical University between December 11, 2016 to August 25, 2017. These patients were screened for enrollment by following inclusion criteria, i) Patients were eligible for the study if they were receiving antimicrobial therapy and if their expected length of stay was more than 2 days; ii) patients who were willing to participate in the study. We recorded age, sex, reason for admission, and receipt of antibiotics. *C. difficile* infection (CDI) was defined as hospital-associated diarrhea (HAD) with a positive stool for *C. difficile* isolation. Asymptomatic patient was defined as a positive stool for *C. difficile* isolation without HAD [9].

**Informed consent**: Informed consent has been obtained from all individuals included in this study.

**Ethical approval**: The research related to human use has been complied with all the relevant national regulations, institutional policies and in accordance the tenets of the Helsinki Declaration, and has been approved by the Human Ethics Committee of Guizhou Medical University (approval no. 2017-004).

### 2.2 Sample collection and processing

Stool samples were collected in 50 ml DNase & RNase-free NEST^®^ sample collection tube (Nest Scientific USA, Inc.) and transferred to the laboratory immediately on ice. The samples were soaked in the appropriate amount of fresh BHI medium for 10 min and vortexed for 10-20 sec. The mixed solution was serially diluted in fresh BHI medium and spread across BHI-blood or CCFA-blood (Cycloserine-Cefoxitin-Fructose Agar, Oxoid) agar [10]. The plates were incubated in an anaerobic chamber at 37˚C for 48 h [11]. Colonies were picked and further purified by re-streaking on a BHI-blood agar plate.

### 2.3 16S rDNA sequencing and phylogenetic analysis

The genomic DNA of purified strains were prepared using a TIANGEN^®^ bacterial genomic DNA extraction kit (DP302; TIANGEN Biotech, Beijing, China). Primers for 16s-V4-515F (5’-GTGCCAGCMGCCGCGGTAA-3’) and 16S-V4-806R (5’-GGACTCHVGGGT-WTCTAAT-3’) were used to amplify partial 16S rDNA of isolated strains according to Lianbing Lin et al. [12]. PCR amplification was performed in a GeneAmp^®^ PCR system 9700 (Applied Biosystems; Thermo Fisher Scientific, Inc., Waltham, MA, USA) using Q5^®^ High-Fidelity Polymerase. The thermocycling conditions were as follows: 98°C for 30 sec; 30 cycles of 98°C for 10 sec, 55°C for 30 sec and 72°C for 10 sec; and a final extension at 72°C for 2 min. The PCR amplification products were recovered directly by TIANGEN^®^ PCR purification kit (DP204, TIANGEN Biotech). A total of 20 μl molecular grade water (heated to 60°C prior to applying to the column) was used to elute purified 16S rDNA. The DNA samples were stored at -20°C prior to sequencing by GeneCreate Biotech (Wuhan, China). Sequencing primers were the same as the primers used in 16S rDNA amplification.

The sequencing results of both directions were assembled using SeqMan software of Lasergene (DNAStar, Madison, WI, USA). The partial 16S rDNA sequences were blasted against the 16SMicrobial database (ftp://ftp. ncbi.nlm.nih.gov/blast/db/v5) using the NCBI-blast-2.7.1 algorithm (ftp://ftp.ncbi.nlm.nih.gov/blast/executables/blast+/2.8.0alpha) and analyzed by Molecular Evolutionary Genetics Analysis version 7 (MEGA7) [13]. For phylogenetic analysis, the evolutionary history was determined using the Maximum Likelihood method based on the Tamura-Nei model [14]. Evolutionary analyses were conducted in MEGA7 [13].

Initial tree(s) for the heuristic search were obtained automatically by applying Neighbor-Join and BioNJ algorithms to a matrix of pairwise distances estimated using the Maximum Composite Likelihood (MCL) approach and then selecting the topology with superior log-likelihood value. The tree is drawn to scale. The analysis involved 23 nucleotide sequences. All positions containing gaps and missing data were eliminated. A total of 230 positions were identified in the final dataset. Evolutionary analyses were conducted in MEGA7 [13].

## 3 Results

### 3.1 Patient characteristics

A total of 51 patients were enrolled in the study during their hospitalization in the affiliated hospital of Guizhou Medical University from 11/12/2016 to 25/8/2017. Among these patients, one patient developed CDI; two patients were confirmed to exhibit asymptomatic *C. difficile* colonization (*C. difficile* was isolated from his/her stool samples, however, the patients did not develop any CDI symptoms, such as diarrhea and megacolon, Tables 1 and 2). The patient with CDI experienced diarrhea, which was not recurrent following antibiotic treatment. Patient characteristics are presented in Tables 1 and 2. The incident rate of CDI was nearly 2%, and incident rate of asymptomatic *C. difficile* colonization was nearly 4%.

**Table 1:** Patient clinicopathological characteristics

Variable	Neither *C. difficile* infection nor colonization (n=51)	*C. difficile* infection (n=1)	*C. difficile* colonization (n=2)	
Age, mean years (range)	56 (18-90)	71	58	70
Sex, Male (Female)	35 (16)	1 (0)	1 (0)	0 (1)
Duration of hospitalization, median days (range)^a^	7 (5-21)	8	6	6
**Reason for admission**				
Pneumonia	17 (33%)	0	1 (50%)	0
Respiratory failure	4 (8%)	0	0	0
Brain Injury	12 (24%)	0	0	0
Cerebral	1 (2%)	0	0	0
Pancreatitis	2 (4%)	0	0	0
Cholangitis	1 (2%)	1 (100%)	0	0
Gastrointestinal bleeding	4 (8%)	0	0	0
Intestinal obstruction	2 (4%)	0	0	1 (50%)
Epilepsy	1 (2%)	0	0	0
Renal failure	1 (2%)	0	0	0
Myocardial infarction	1 (2%)	0	0	0
Others	5 (10%)	0	0	0
**Antibiotic usage** ^b^				
Cefuroxime	12 (24%)	0	0	0
Cefoperazone sodium sulbactam sodium	31 (61%)	1 (100%)	0	0
Tinidazole	14 (27%)	1 (100%)	0	0
Meropenem	13 (25%)	0	0	0
Imipenem cilastatin	15 (29%)	0	0	0
Vancomycin	7 (14%)	0	0	0
Fluconazole	14 (22%)	0	0	0
Mikafen	11 (22%)	0	0	0
Tigecycline	10 (20%)	0	0	0
Gentamicin	1 (2%)	0	0	0
Linezolid	6 (12%)	0	0	0
Levofloxacin	2 (4%)	0	0	0
Moxifloxacin	3 (6%)	0	0	0
Voriconazole	6 (12%)	0	0	0
Isoniazid	2 (4%)	0	0	0
Rifampin	2 (4%)	0	0	0
Ethambutol	2 (4%)	0	0	0
Pyrazinamide	1 (2%)	0	0	0
Ceftazidime	1 (2%)	0	1 (50%)	0
Cefmetazole sodium	1 (2%)	0	0	0
Piperacillin-tazobactam sodium	4 (8%)	0	0	1 (50%)
Oxacillin sodium	1 (2%)	0	0	0

a From admission until diagnosis of *C. difficile* infection or colonization (for patients with CDI and asymptomatic *C. difficile* colonization) or until discharge (for patients without infection or colonization). ^b^ Antibiotic usage before confirming of CDI infection or colonization.

**Table 2:** Detection of *C*. *difficile* in patients with asymptomatic colonization and CDI.

Patient ID	Sample type	Culture	Medication
Asymptomatic colonization			
37	Stool	Positive	Ceftazidime
42	Stool	Positive	Piperacillin-tazobactam sodium
CDI			
41	Stool	Positive	Tinidazole & Cefoperazone sodium sulbactam sodium

CDI, *Clostridium difficile* infection.

### 3.2 Isolation of bacteria that co-colonize with *C. difficile*

**Figure 1:**
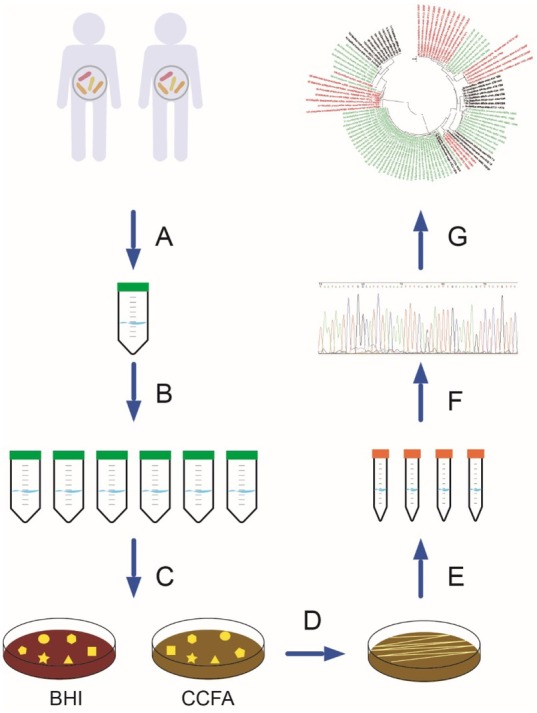
Schematic representation of the experimental design. (A) Stool samples from two patients with asymptomatic *C. difficile* coloization were collected in sterile 50 ml tubes and transferred to the lab on ice. (B) Samples were resuspended in 10 ml fresh BHIS medium and serially diluted 10 times. (C) 100 μl serial diluted samples were plated on BHI-blood and CCFA-blood medium and incubated anaerobically at 37^o^C for 24-48 h until colonies formed. (D) A loopful of each colony was re-streaked on either BHI-blood or CCFA-blood medium for further purification. (E) Purified colonies were inoculated in 5 ml BHI-blood or CCFA-blood broth and inoculated in an anaerobic chamber for 24 h. The resulting strain culture was subjected to genomic DNA extraction. (F) Genomic DNA of each strain was used as a template in the PCR to amplify the 16S rDNA. Partial 16S rDNA samples were sent to GeneCreat Biotech for further DNA sequencing. (G) 16S rDNA sequences were analyzed by Molecular Evolutionary Genetics Analysis version 7 software.

The stool samples of asymptomatic patients were analyzed using BHIS-blood and CCFA-blood medium without antibiotics (Figure 1) [15, 16]. A total of 111 strains were isolated from the two fecal samples. The strains were purified, and the partial 16S ribosomal gene sequences were obtained and blasted against the 16SMicrobial database. The blast results are presented in Table 3. Among the strains, *Escherichia* species (*E. marmotae* and *E. fergusonii*; n=37; 33.3%) was the most abundant species co-colonizing with *C. difficile*. *Enterococcus* (*E. saigonensis, E. faecalis, E. hirae*; n=13; 11.7%) and *Clostridium* (*C. clostridioforme*, *C. tertium*; n=12; 10.8%) were ranked in the second and third place, respectively (Table 3).

**Table 3:** Strain identification by the partial 16S ribosomal gene^a^.

*Strain*	Strain isolated (Percentage of all isolates, %)	Phylum	Class	Score (Bits)	E Value
*.Escherichia marmotae*	33 (30)	*Proteobacteria*	*Gammaproteobacteria*	387	2.00E-107
*Clostridium difficile*	15 (14)	*Firmicutes*	*Clostridia*	2102	0.00E+00
*Clostridium clostridioforme*	11 (10)	*Firmicutes*	*Clostridia*	429	3.00E-120
*Enterococcus saigonensis*	5 (5)	*Firmicutes*	*Bacilli*	403	2.00E-112
*Ruminococcus gnavus*	5 (5)	*Firmicutes*	*Clostridia*	420	2.00E-117
*Enterococcus faecalis*	5 (5)	*Firmicutes*	*Bacilli*	2193	0.00E+00
*Escherichia fergusonii*	4 (4)	*Firmicutes*	*Bacilli*	436	2.00E-122
*Enterococcus hirae*	3 (3)	*Firmicutes*	*Bacilli*	771	0
*Klebsiella pneumoniae*	3 (3)	*Proteobacteria*	*Gammaproteobacteria*	760	0.00E+00
*Klebsiella quasipneumoniae*	4 (4)	*Proteobacteria*	*Gammaproteobacteria*	778	0.00E+00
*Fusobacterium nucleatum*	4 (4)	*Fusobacteriia*	*Fusobacteriales*	422	6.00E-118
*Ralstonia pickettii*	3 (3)	*Proteobacteria*	*Proteobacteria*	403	2.00E-112
*Bacillus tropicus*	2 (2)	*Firmicutes*	*Bacilli*	765	0
*Shigella dysenteriae*	2 (2)	*Proteobacteria*	Gammaproteobacteria	379	3.00E-105
*Lactobacillus paracasei*	3 (3)	*Firmicutes*	*Bacilli*	414	9.00E-116
*Pseudochrobactrum lubricantis*	2 (2)	*Proteobacteria*	*Alphaproteobacteria*	418	8.00E-117
*Blautia producta*	1 (1)	*Firmicutes*	*Clostridia*	436	2.00E-122
*Veillonella parvula*	1 (1)	*Firmicutes*	*Negativicutes*	438	6.00E-123
*Acinetobacter baumannii*	1 (1)	*Proteobacteria*	*Gammaproteobacteria*	773	0
*Bacillus cereus*	1 (1)	*Firmicutes*	*Bacilli*	2141	0.00E+00
*Fusobacterium simiae*	1 (1)	*Fusobacteriia*	*Fusobacteriales*	427	7.00E-117
*Herbaspirillum chlorophenolicum*	1 (1)	*Proteobacteria*	*Betaproteobacteria*	424	2.00E-118
*Clostridium tertium*	1 (1)	*Firmicutes*	*Clostridia*	719	0

a Green represents strains that have been reported as normal human habitats and may act as protective taxa against CDI. Red represents human pathogens that co-colonize with *C. difficile*. Black represents bacteria of which the pathogenicity to human is unknown (except *C. difficile*).

### 3.3 Phylogenetic analyses

**Figure 2:**
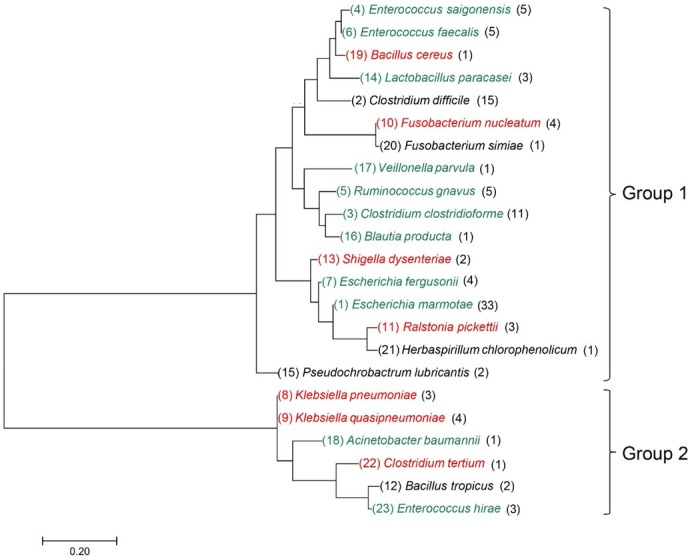
Molecular phylogenetic analysis by Maximum Likelihood method. The evolutionary history was determined using the Maximum Likelihood method based on the Tamura-Nei model [14]. The isolated strain number for each species is indicated on the left. Green indicates strains that have been reported as normal human habitats and may act as protective taxa against CDI. Red represents human pathogens cocolonizing with *C. difficile*. Black represents strains of which the pathogenicity to humans is unknown (except *C. difficile*). The tree with the highest log likelihood (-2403.04) is presented.

**Figure S1:**
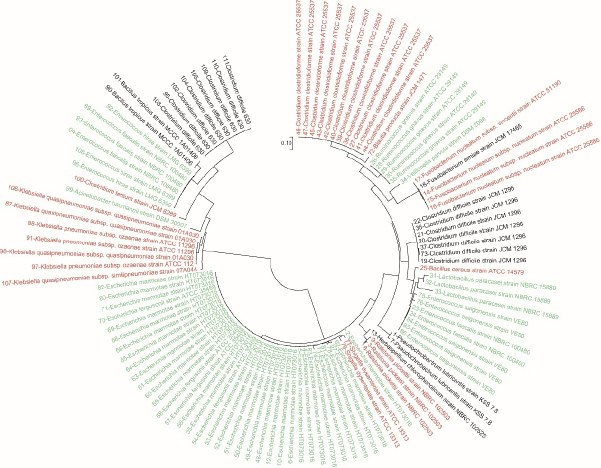
**Phylogenetic tree of the 111 isolated strains that co-colonized with** C. difficile **in asymptomatic patients**. The evolutionary history was determined using the Maximum Likelihood method based on the Tamura-Nei model [14]. Green represents strains that have been reported as normal human habitats and may act as protective taxa against CDI. Red represents human pathogens co-colonizing with *C. difficile*. Black represents bacteria of which the pathogenicity to humans is unknown (except *C. difficile*).

To determine the taxonomic associations of the isolated strains, a phylogenomic tree was constructed based on the values of nucleotide sequence pairwise similarity between the isolates. Strains from same species were clustered together (Figure S1). To provide an in-depth view of the association between isolated species, duplicated strains were omitted and the actual number of isolated strains was subsequently marked (Figure 2). A total of 22 types of strains were demonstrated to co-colonize with *C. difficile*. The strains were grouped into two major groups: Group 1 and group 2 (Figure 2). The majority of strains in Group 1 belonged to the *Firmicutes* phylum, whereas the majority of strains in group 2 belonged to the *Proteobacteria* phylum. Bacteria from the *Bacteroidete* phylum was not isolated in the two samples [8]. This may have been caused by the bias of the medium or the novel gut microbiota structure of the patients, which may be affected by specific dietary habits and antibiotic use.

Two types of *C. difficile* strains were isolated from the stool samples, which mostly related to the *C. difficile* JCM 1296 and *C. difficile* 630 strain (Figure S1). Among all strains, 51.3% (57/111) of the isolated strains belonged to the *Firmicutes* phylum, which contained three classes: *Clostridia* (57.9%, 33/57), *Bacilli* (40.4%, 23/57) and *Negativicutes* (1.8%, 1/55); 45.9% (49/111) of the isolates belonged to the *Proteobacteria* phylum, which contained four classes: *Gammaproteobacteria* (87.8%, 43/49), *Proteobacteria* (6.1%, 3/49), *Alphaproteobacteria* (4.1%, 2/49) and *Betaproteobacteria* (2%, 1/49). In the *Fusobacteriia* phylum (4.5%, 5/111), *Fusobacteriales* was the only isolated class. To the best of our knowledge, this was the first time that *Fusobacteriia* was co-isolated with *C. difficile*, which was not previously demonstrated in metagenomic research [8].

## 4 Discussion

The present study used the culture method to analyze the microbial diversity in the stool samples of two asymptomatic patients with *C. difficile* colonization. A total of 111 strains were isolated from these samples, their partial 16S ribosome genes were sequenced, and NCBI-blast-2.7.1 and MEGA7 algorithms were used to determine the diversity and phylogenetic associations of these isolates. The isolates were derived from three phyla: *Firmicutes*, *Proteobacteria* and *Fusobacteriia*. *Firmicutes* (51.3%) and *Proteobacteria* (44.1%) were most abundant phyla. To the best of our knowledge, this is the first time that *Fusobacteriia* (4.5%) was reported to co-colonize with *C. difficile*. Comparing with metagenomic research [8], *Bacteroidete* phylum was not identified in the present study, which may be due to the bias of screening medium and antibiotics used during the hospitalization of patients.

Although metagenomic sequencing approaches can provide abundant data for culturable and nonculturable microorganisms, culturomics has become increasingly important in recent years [17], as it may enable the design of a defined microbiota composition, which may be transferred to patients with CDI by FMT. Ann M.O’Hara *et al* have suggested that the microbial composition of the gut contributes to intestinal disorders and that the enhancement of beneficial bacteria may represent a promising therapeutic strategy against various diseases (e.g., CDI) caused by disruptions in the gut microbiota [18]. The present study demonstrated that *Escherichia* (33.3%), *Clostridium* (24.3%) and *Enterococcus* (11.7%) exhibited high ratios in the two tested samples, which was consistent with previous research [19]. These species may serve as protective taxa against the transition from asymptomatic *C. difficile* colonization to CDI. For example, *Clostridium* spp. are potential protective bacterial taxa that may exert their protective effects through the production of secondary bile acids [8]; *Lactobacillus paracasei* strains have been demonstrated to exhibit health-promoting properties as probiotics [19, 20]. Recently, *Blautia producta*, *Ruminococcus*, *Lactobacillus paracasei* and *Escherichia* have been used in a defined stool substitute mixture to treat antibiotic-resistant *C. difficile* colitis [21].

By contrast, species that are normally considered human pathogens were also identified to co-colonize with *C. difficile*, including *Fusobacterium nucleatum*, *Bacillus cereus*, *Shigella dysenteriae*, *Ralstonia pickettii*, *Klebsiella pneumoniae*, *Klebsiella quasipneumoniae* and *Clostridium tertium*. *Fusobacterium nucleatum* normally colonizes in the oral environment and has recently been demonstrated to be associated with intestinal tumorigenesis [22]. *Bacillus cereus* is easily transferred through food and may cause emetic or diarrheal food-associated illness [23]. *Shigella dysenteriae* causes dysentery, which occurs most frequently in areas where poor sanitation and malnutrition are prevalent, especially in developing countries [24]. *Klebsiella quasipneumoniae* has been reported to cause pyogenic liver abscess [25]. *Clostridium tertium* commonly affects neutropenic patients with haematological malignancy [26]. In addition, to the best of our knowledge, *Fusobacterium nucleatum*, *Ralstonia pickettii*, *Klebsiella pneumoniae*, *Clostridium tertium* and *Klebsiella quasipneumoniae* have not been reported to co-infect with *C. difficile* [27]. These results suggested that co-infection may be ubiquitous during CDI progression. In this case, it could be associated to the special dietary habits in the Guizhou province, where pickled and spicy food is preferred. However, the underlying mechanism needs to be studied further.

In the two asymptomatic patients, *C. difficile* was detected by culturing method. Due to the number of asymptomatic patients in present study was limited and the microbiota composition is strongly influenced by their illness and medical treatment [8]. Therefore, we could not perform statistical analyses to assess general abundance of microbial taxa for asymptomatic patients. Furthermore, there are still two concerns should be carefully addressed in future studies. Firstly, the diversity of microbes was relatively low in the present study. For instance, *Bacteroidete*, *Virus* and *Fungi* were not identified; this may have been due to the bias of the screening medium and/or antibiotic use of the patients during hospitalization. These problems should be carefully addressed in future studies. Secondly, co-colonization may also increase the potential for genetic transference of resistance, which results in the development of antibiotic-resistant pathogens [28]. However, weather horizontal transfer of antibiotic resistance-associated genes occurs among the isolated species is largely unknown.

In summary, the present study used the culture method to analyze stool samples from two patients with asymptomatic *C. difficile* colonization in Guizhou province. This is the first report of microbial diversity in *C. difficile* carriers in southwest China, where specific dietary habits are prevalent, with a preference for pickled and spicy food. The results of the present study may improve the awareness of CDI among clinicians and provide new options for CDI treatment in southwest China.
